# Supplementary material for: Does age of ADHD medication initiation predict long-term risk of anxiety? A scoping review
Source: PLOS Ment Health. 2025 Jan 10;2(1):e0000230. doi: 10.1371/journal.pmen.0000230 (PMC12798288; doi:10.1371/journal.pmen.0000230)
Supplement: S2 Table — (DOCX) [file pmen.0000230.s002.docx]

**S2 Table.**

**Librarian Searcher:** Leila Ledbetter MLIS, AHIP, Duke University Medical Center Library

**Original Search Date:** July 17, 2023

Update Search Dates: July 10, 2024

**Database / Study Registry (including vendor/platform):** Medline (via PubMed), Inclusive Date Coverage: 1966 to present

| Set # |  | Results |
| --- | --- | --- |
| 1 ADD/ADHD | "Attention Deficit Disorder with Hyperactivity"[Mesh] OR "Impulsive Behavior"[Mesh] OR "Psychomotor Agitation"[MeSH] OR "Attention"[Mesh] OR "Arousal"[Mesh] OR ADHD[tiab] OR ADDH[tiab] OR ADD[tiab] OR "ADD-ADHD"[tiab] OR ADD/ADHD[tiab] OR "attention-deficit"[tiab] OR hyperactive[tiab] OR hyperactivity[tiab] OR Hyperactiveness[tiab] "hyper activity"[tiab] OR restless[tiab] OR restlessness[tiab] OR "attention deficit"[tiab] OR attentive[tiab] OR inattention[tiab] OR inattentive[tiab] OR distractible[tiab] OR distractibility[tiab] OR distracted[tiab] OR impulsive[tiab] OR impulsiveness[tiab] OR impulsivity[tiab] OR impulsivities[tiab] OR arousal[tiab] OR attentional[tiab] OR forgetful[tiab] OR Hyperkinetic[tiab] OR "Executive function"[tiab] OR "Executive functioning"[tiab] OR "Sluggish cognitive tempo"[tiab] OR "cognitive disengagement"[tiab] | 170,022 |
| 2  Child/adolescent | "Child"[Mesh] OR "Adolescent"[Mesh] OR "Pediatrics"[Mesh] OR "Minors"[Mesh] OR kid[tiab] OR kids[tiab] OR child[tiab] OR childhood[tiab] OR children[tiab] OR preadolescent[tiab] OR preadolescents[tiab] OR preadolescence[tiab] OR adolescent[tiab] OR adolescents[tiab] OR adolescence[tiab] OR juvenile[tiab] OR juveniles[tiab] OR youth[tiab] OR youths[tiab] OR teen[tiab] OR teens[tiab] OR teenager[tiab] OR teenagers[tiab] OR teenaged[tiab] OR pediatric[tiab] OR pediatrics[tiab] OR paediatric[tiab] OR paediatrics[tiab] OR minor[tiab] OR minors[tiab] OR youth[tiab] OR youths[tiab] | 4,262,410 |
| 3  Medication | "methylphenidate"[MeSH Terms] OR "methylphenidate"[tiab] OR "methylphenidate s"[tiab] OR "methylphenidates"[tiab] OR "dexmethylphenidate hydrochloride"[MeSH Terms] OR "dexmethylphenidate hydrochloride"[tiab] OR "dexmethylphenidate"[tiab] OR "serdexmethylphenidate"[tiab] OR "concerta"[tiab] OR "cotempla"[tiab] OR "ritalin"[tiab] OR "ritaline"[tiab] OR "ritalinic"[tiab] OR "methylin"[tiab] OR "Medikinet"[tiab] OR "equasym"[tiab] OR "quillivant"[tiab] OR "metadate"[tiab] OR "dextroamphetamine"[MeSH Terms] OR "dextroamphetamine"[tiab] OR dexamphetamine[tiab] OR "dexedrine"[tiab] OR "procentra"[tiab] OR "zenzedi"[tiab] OR "amphetamine s"[tiab] OR "amphetamines"[MeSH Terms] OR "amphetamines"[tiab] OR "amphetaminic"[tiab] OR "amphetamine"[tiab] OR "amphetamine"[MeSH Terms] OR "adderall"[Supplementary Concept] OR "adderall"[tiab] OR "lisdexamfetamine dimesylate"[MeSH Terms] OR "lisdexamfetamine dimesylate"[tiab] OR "lisdexamfetamine"[tiab] OR "vyvanse"[tiab] OR "Venvanse"[tiab] OR "elvanse"[tiab] OR "Tyvense"[tiab] OR "atomoxetine hydrochloride"[MeSH Terms] OR "atomoxetine hydrochloride"[tiab] OR "atomoxetine"[tiab] OR "atomoxetine s"[tiab] OR "strattera"[tiab] OR "guanfacine"[MeSH Terms] OR "guanfacine"[tiab] OR "intuniv"[tiab] OR "estulic"[tiab] OR "tenex"[tiab] OR "clonidine"[MeSH Terms] OR "clonidine"[tiab] OR "clonidin"[tiab] OR "clonidine s"[tiab] OR "catapres"[tiab] OR "clopheline"[tiab] OR "clophelin"[tiab] OR "kapvay"[tiab] OR "nexiclon"[tiab] OR "duraclon"[tiab] OR "desipramine"[MeSH Terms] OR "desipramine"[tiab] OR "norpramin"[tiab] OR "desipramine s"[tiab] OR "pertofrane"[tiab] OR "buproprion"[tiab] OR "bupropion"[MeSH Terms] OR "amfebutamone"[tiab] OR "wellbutrin"[tiab] OR "bupropion s"[tiab] OR "bupropione"[tiab] OR "zyban"[tiab] OR "modafinil"[MeSH Terms] OR "modafinil"[tiab] OR "modafinil s"[tiab] OR "provigil"[tiab] OR "alertec"[tiab] OR "modiodal"[tiab] OR "Modalert"[tiab] OR "armodafinil"[tiab] OR "nuvigil"[tiab] OR "venlafaxin"[tiab] OR "venlafaxine hydrochloride"[MeSH Terms] OR "venlafaxine hydrochloride"[tiab] OR "venlafaxine"[tiab] OR "venlafaxine hydrochloride"[MeSH Terms] OR "effexor"[tiab] OR "trevilor"[tiab] OR "duloxetin"[tiab] OR "duloxetine hydrochloride"[MeSH Terms] OR "duloxetine hydrochloride"[tiab] OR "duloxetine"[tiab] OR "duloxetine s"[tiab] OR "duloxetin"[tiab] OR "cymbalta"[tiab] OR "selegiline"[MeSH Terms] OR "selegiline"[tiab] OR "selegiline s"[tiab] OR "eldepryl"[tiab] OR "emsam"[tiab] OR "Selgene"[tiab] OR "zelapar"[tiab] OR "amantadin"[tiab] OR "amantadine"[MeSH Terms] OR "amantadine"[tiab] OR "amantadines"[tiab] OR "symmetrel"[tiab] OR "mematine"[tiab] OR "memantine"[MeSH Terms] OR "memantin"[tiab] OR "namenda"[tiab] OR "memantine s"[tiab] OR "daytrana"[tiab] OR "focalin"[tiab] OR "jornay"[tiab] OR "adzenys"[tiab] OR "methamphetamine"[MeSH Terms] OR "methamphetamine"[tiab] OR "desoxyn"[tiab] OR "methamphetamine s"[tiab] OR "methamphetamines"[tiab] OR "dynavel"[tiab] OR "evekeo"[tiab] OR "mydayis"[tiab] OR "qelbree"[tiab] OR "viloxazin"[tiab] OR "viloxazine"[MeSH Terms] OR "viloxazine"[tiab] OR psychotropic[tiab] OR psychotropics[tiab] OR psychostimulant[tiab] OR psychostimulants[tiab] OR psychopharmacological[tiab] OR psychopharmacologicals[tiab] OR medication[tiab] OR medications[tiab] OR pharmacological[tiab] OR pharmacologics[tiab] OR pharmacologically[tiab] OR pharmacologic[tiab] OR stimulant[tiab] OR stimulants[tiab] OR “non-stimulant”[tiab] OR “non-stimulants”[tiab] | 137,419 |
| 4 Anxiety | "Anxiety Disorders"[MeSH Terms] OR "Anxiety"[MeSH Terms] OR "Anxiety"[tiab] OR "anxieties"[tiab] OR "anxiety s"[tiab] OR "anxious"[tiab] OR "anxiousness"[tiab] OR "nervousness"[tiab] OR "nervous"[tiab] OR "worried"[tiab] OR "worries"[tiab] OR "worry"[tiab] OR "worrying"[tiab] OR "worrisome"[tiab] OR "distress"[tiab] OR "distressed"[tiab] OR "distresses"[tiab] OR "distressful"[tiab] OR "distressing"[tiab] OR "stress"[tiab] OR "stressed"[tiab] OR "stresses"[tiab] OR "stressful"[tiab] OR "stressfulness"[tiab] OR "stressing"[tiab] OR "uncertainty"[MeSH Terms] OR "uncertainty"[tiab] OR "insecure"[tiab] OR "insecurely"[tiab] OR "insecurities"[tiab] OR "insecurity"[tiab] OR "angst"[tiab] OR "hypervigilance"[tiab] OR "hypervigilant"[tiab] OR "panic"[MeSH Terms] OR "panic"[tiab] OR "panics"[tiab] OR "neurotic"[tiab] OR "neurotically"[tiab] OR "neurotics"[tiab] OR Neurosis[tiab] OR Neuroses[tiab] OR Psychoneuroses[tiab] OR "Phobic Disorders"[Mesh] OR Phobia[tiab] OR Phobic[tiab] OR "Agoraphobia"[tiab] OR "Panic Disorder"[Mesh] | 1,951,584 |
| 5 | #1 and #2 and #3 AND #4 | 803 |
| 6  Cohort and case-series Study types | “Case-Control Studies”[Mesh:noexp] OR "retrospective studies"[mesh:noexp] OR “Control Groups”[Mesh:noexp] OR (case[TIAB] AND control[TIAB]) OR (cases[TIAB] AND controls[TIAB]) OR (cases[TIAB] AND controlled[TIAB]) OR (case[TIAB] AND comparison*[TIAB]) OR (cases[TIAB] AND comparison*[TIAB]) OR “control group”[TIAB] OR “control groups”[TIAB] OR “cohort studies”[mesh:noexp] OR “longitudinal studies”[mesh:noexp] OR “follow-up studies”[mesh:noexp] OR “prospective studies”[mesh:noexp] OR “retrospective studies”[mesh:noexp] OR cohort[TIAB] OR longitudinal[TIAB] OR prospective[TIAB] OR retrospective[TIAB] OR “Cross-Sectional Studies”[Mesh:noexp] OR “cross-sectional”[TIAB] OR Prevalence[mesh:noexp] OR prevalence[tiab] OR transversal study[tiab] OR lifespan[tiab] | 5,276,507 |
| 7 | #5 AND #6 | 648 |
| 8 | #7 NOT ("Adult"[Mesh] NOT ("Adolescent"[Mesh] OR "Child"[Mesh] OR "Infant"[Mesh])) | 623 |
| 9 | #8 NOT (Editorial[ptyp] OR Letter[ptyp] OR Comment[ptyp]) NOT (animals[mh] NOT humans[mh]) | 617 |

**Database / Study Registry:** Embase (Elsevier), Inclusive Date Coverage: 1947 - present

| Set # |  | Results |
| --- | --- | --- |
| 1 ADD/ADHD | 'attention deficit hyperactivity disorder'/de OR 'impulsiveness'/de OR 'disruptive behavior'/de OR 'restlessness'/de OR 'Attention'/de OR 'Arousal'/de OR 'sluggish cognitive tempo'/de OR ADHD:ti,ab OR ADDH:ti,ab OR ADD:ti,ab OR 'ADD-ADHD':ti,ab OR 'ADD':ti,ab OR 'attention-deficit':ti,ab OR hyperactive:ti,ab OR hyperactivity:ti,ab OR restless:ti,ab OR restlessness:ti,ab OR 'attention deficit':ti,ab OR attentive:ti,ab OR inattention:ti,ab OR inattentive:ti,ab OR distractible:ti,ab OR distractibility:ti,ab OR distracted:ti,ab OR impulsive:ti,ab OR impulsiveness:ti,ab OR impulsivity:ti,ab OR impulsivities:ti,ab OR arousal:ti,ab OR attentional:ti,ab OR forgetful:ti,ab OR Hyperkinetic:ti,ab OR 'Executive function':ti,ab OR 'Executive functioning':ti,ab OR 'Sluggish cognitive tempo':ti,ab OR 'cognitive disengagement':ti,ab | 571,591 |
| 2  Child/adolescent | Child/de OR Adolescent/de OR Pediatrics/de OR 'minor (person)'/de OR kid:ti,ab OR kids:ti,ab OR child:ti,ab OR childhood:ti,ab OR children:ti,ab OR preadolescent:ti,ab OR preadolescents:ti,ab OR preadolescence:ti,ab OR adolescent:ti,ab OR adolescents:ti,ab OR adolescence:ti,ab OR juvenile:ti,ab OR juveniles:ti,ab OR youth:ti,ab OR youths:ti,ab OR teen:ti,ab OR teens:ti,ab OR teenager:ti,ab OR teenagers:ti,ab OR teenaged:ti,ab OR pediatric:ti,ab OR pediatrics:ti,ab OR paediatric:ti,ab OR paediatrics:ti,ab OR minor:ti,ab OR minors:ti,ab | 4,675,386 |
| 3  Medication | methylphenidate/de OR methylphenidate:ti,ab OR 'methylphenidate s':ti,ab OR methylphenidates:ti,ab OR 'dexmethylphenidate'/de OR 'dexmethylphenidate hydrochloride':ti,ab OR dexmethylphenidate:ti,ab OR serdexmethylphenidate:ti,ab OR concerta:ti,ab OR cotempla:ti,ab OR ritalin:ti,ab OR ritaline:ti,ab OR ritalinic:ti,ab OR methylin:ti,ab OR Medikinet:ti,ab OR equasym:ti,ab OR quillivant:ti,ab OR metadate:ti,ab OR 'dexamphetamine'/de OR dexamphetamine:ti,ab OR dextroamphetamine:ti,ab OR dexedrine:ti,ab OR procentra:ti,ab OR zenzedi:ti,ab OR 'amphetamine s':ti,ab OR 'amphetamine derivative'/de OR amphetamines:ti,ab OR amphetaminic:ti,ab OR amphetamine:ti,ab OR amphetamine/de OR adderall:tn OR adderall:ti,ab OR 'lisdexamfetamine'/de OR 'lisdexamfetamine dimesylate':ti,ab OR lisdexamfetamine:ti,ab OR vyvanse:ti,ab OR Venvanse:ti,ab OR elvanse:ti,ab OR Tyvense:ti,ab OR 'atomoxetine'/de OR 'atomoxetine hydrochloride':ti,ab OR atomoxetine:ti,ab OR 'atomoxetine s':ti,ab OR strattera:ti,ab OR guanfacine/de OR guanfacine:ti,ab OR intuniv:ti,ab OR estulic:ti,ab OR tenex:ti,ab OR clonidine/de OR clonidine:ti,ab OR clonidin:ti,ab OR 'clonidine s':ti,ab OR catapres:ti,ab OR clopheline:ti,ab OR clophelin:ti,ab OR kapvay:ti,ab OR nexiclon:ti,ab OR duraclon:ti,ab OR desipramine/de OR desipramine:ti,ab OR norpramin:ti,ab OR 'desipramine s':ti,ab OR pertofrane:ti,ab OR buproprion:ti,ab OR 'amfebutamone'/de OR amfebutamone:ti,ab OR wellbutrin:ti,ab OR 'bupropion s':ti,ab OR bupropione:ti,ab OR zyban:ti,ab OR modafinil/de OR modafinil:ti,ab OR 'modafinil s':ti,ab OR provigil:ti,ab OR alertec:ti,ab OR modiodal:ti,ab OR Modalert:ti,ab OR armodafinil:ti,ab OR nuvigil:ti,ab OR venlafaxin:ti,ab OR 'venlafaxine'/de OR 'venlafaxine hydrochloride':ti,ab OR venlafaxine:ti,ab OR effexor:ti,ab OR trevilor:ti,ab OR duloxetin:ti,ab OR 'duloxetine'/de OR 'duloxetine hydrochloride':ti,ab OR duloxetine:ti,ab OR 'duloxetine s':ti,ab OR duloxetin:ti,ab OR cymbalta:ti,ab OR selegiline/de OR selegiline:ti,ab OR 'selegiline s':ti,ab OR eldepryl:ti,ab OR emsam:ti,ab OR Selgene:ti,ab OR zelapar:ti,ab OR amantadin:ti,ab OR amantadine/de OR amantadine:ti,ab OR amantadines:ti,ab OR symmetrel:ti,ab OR mematine:ti,ab OR memantine/de OR memantin:ti,ab OR namenda:ti,ab OR 'memantine s':ti,ab OR daytrana:ti,ab OR focalin:ti,ab OR jornay:ti,ab OR adzenys:ti,ab OR methamphetamine/de OR methamphetamine:ti,ab OR desoxyn:ti,ab OR 'methamphetamine s':ti,ab OR methamphetamines:ti,ab OR dynavel:ti,ab OR evekeo:ti,ab OR mydayis:ti,ab OR qelbree:ti,ab OR viloxazin:ti,ab OR viloxazine/de OR viloxazine:ti,ab OR psychotropic:ti,ab OR psychotropics:ti,ab OR psychostimulant:ti,ab OR psychostimulants:ti,ab OR psychopharmacological:ti,ab OR psychopharmacologicals:ti,ab OR medication:ti,ab OR medications:ti,ab OR pharmacological:ti,ab OR pharmacologics:ti,ab OR pharmacologically:ti,ab OR pharmacologic:ti,ab OR stimulant:ti,ab OR stimulants:ti,ab OR 'non-stimulant':ti,ab OR non-stimulants:ti,ab | 1,381,746 |
| 4 Anxiety | 'Anxiety Disorder’/de OR Anxiety/de OR Anxiety:ti,ab OR anxieties:ti,ab OR 'anxiety s':ti,ab OR anxious:ti,ab OR anxiousness:ti,ab OR nervousness:ti,ab OR nervous:ti,ab OR worried:ti,ab OR worries:ti,ab OR worry:ti,ab OR worrying:ti,ab OR worrisome:ti,ab OR distress:ti,ab OR distressed:ti,ab OR distresses:ti,ab OR distressful:ti,ab OR distressing:ti,ab OR stress:ti,ab OR stressed:ti,ab OR stresses:ti,ab OR stressful:ti,ab OR stressfulness:ti,ab OR stressing:ti,ab OR uncertainty/de OR uncertainty:ti,ab OR insecure:ti,ab OR insecurely:ti,ab OR insecurities:ti,ab OR insecurity:ti,ab OR angst:ti,ab OR hypervigilance:ti,ab OR hypervigilant:ti,ab OR panic/de OR panic:ti,ab OR panics:ti,ab OR neurotic:ti,ab OR neurotically:ti,ab OR neurotics:ti,ab OR Neurosis:ti,ab OR Neuroses:ti,ab OR Psychoneuroses:ti,ab OR 'Phobia'/de OR Phobia:ti,ab OR Phobic:ti,ab OR Agoraphobia:ti,ab | 2,565,480 |
| 5 | #1 AND #2 AND #3 AND #4 | 5,272 |
| 6 | 'case control study'/de OR 'retrospective study'/de OR 'controlled study'/de OR 'control group'/de OR 'cohort analysis'/de OR 'longitudinal study'/de OR 'follow up'/de OR 'prospective study'/de OR 'retrospective study'/de OR 'cross-sectional study'/de OR prevalence/de OR 'prevalence study'/de OR lifespan/de OR (case:ti,ab AND control:ti,ab ) OR (cases:ti,ab AND controlled:ti,ab ) OR (cases:ti,ab AND comparison*:ti,ab ) OR 'control group:ti':ab OR 'control groups:ti,ab' OR cohort:ti,ab OR longitudinal:ti,ab OR prospective:ti,ab OR retrospective:ti,ab OR cross-sectional:ti,ab OR prevalence:ti,ab OR 'transversal study:ti,ab' OR lifespan:ti,ab | 14,483,333 |
| 7 | #5 AND #6 | 3,027 |
| 8 | #7 NOT ('Adult'/exp NOT ('Adolescent'/exp OR "Child"/exp)) | 2,777 |
| 9 | #8 NOT ([animals]/lim NOT [humans]/lim) | 2,679 |
| 10 | #9 NOT ('case report'/exp OR 'case study'/exp OR 'editorial'/exp OR [editorial]/lim OR 'letter'/exp OR [letter]/lim OR 'note'/exp OR [note]/lim) | 2,454 |

**Database / Study Registry:** Web of Science (Clarivate) using: Science Citation Index Expanded (SCI-EXPANDED), Social Sciences Citation Index (SSCI), Arts & Humanities Citation Index (A&HCI), Conference Proceedings Citation Index – Science (CPCI-S), Conference Proceedings Citation Index – Social Science & Humanities (CPCI-SSH)

| Set # |  | Results |
| --- | --- | --- |
| 1 ADD/ADHD | TS=("Attention Deficit Disorder with Hyperactivity" OR "Impulsive Behavior” OR "Psychomotor Agitation" OR Attention OR Arousal OR ADHD OR ADDH OR ADD OR "ADD-ADHD" OR ADD OR "attention-deficit" OR hyperactive OR hyperactivity OR restless OR restlessness OR "attention deficit" OR attentive OR inattention OR inattentive OR distractible OR distractibility OR distracted OR impulsive OR impulsiveness OR impulsivity OR impulsivities OR arousal OR attentional OR forgetful OR Hyperkinetic OR "Executive function" OR "Executive functioning" OR "Sluggish cognitive tempo" OR "cognitive disengagement") | 2,580,554 |
| 2  Child/adolescent | TS=( Child OR Adolescent OR Pediatrics OR Minors OR kid OR kids OR childhood OR children OR preadolescent OR preadolescents OR preadolescence OR adolescent OR adolescents OR adolescence OR juvenile OR juveniles OR youth OR youths OR teen OR teens OR teenager OR teenagers OR teenaged OR pediatric OR pediatrics OR paediatric OR paediatrics OR minor OR minors) | 3,340,592 |
| 3  Medication | TS=(methylphenidate OR "methylphenidate s" OR methylphenidates OR "dexmethylphenidate hydrochloride" OR dexmethylphenidate OR serdexmethylphenidate OR concerta OR cotempla OR ritalin OR ritaline OR ritalinic OR methylin OR Medikinet OR equasym OR quillivant OR metadate OR dextroamphetamine OR dexedrine OR procentra OR zenzedi OR "amphetamine s" OR amphetamines OR amphetaminic OR amphetamine OR amphetamine OR adderall OR "lisdexamfetamine dimesylate" OR lisdexamfetamine OR vyvanse OR Venvanse OR elvanse OR Tyvense OR "atomoxetine hydrochloride" OR atomoxetine OR "atomoxetine s" OR strattera OR guanfacine OR intuniv OR estulic OR tenex OR clonidine OR clonidin OR "clonidine s" OR catapres OR clopheline OR clophelin OR kapvay OR nexiclon OR duraclon OR desipramine OR norpramin OR "desipramine s" OR pertofrane OR bupropion OR amfebutamone OR wellbutrin OR "bupropion s" OR bupropione OR zyban OR modafinil OR "modafinil s" OR provigil OR alertec OR modiodal OR Modalert OR armodafinil OR nuvigil OR venlafaxin OR "venlafaxine hydrochloride" OR venlafaxine OR effexor OR trevilor OR duloxetin OR "duloxetine hydrochloride" OR duloxetine OR "duloxetine s" OR duloxetin OR cymbalta OR selegiline OR selegiline OR "selegiline s" OR eldepryl OR emsam OR Selgene OR zelapar OR amantadin OR amantadine OR amantadines OR symmetrel OR mematine OR memantine OR memantin OR namenda OR "memantine s" OR daytrana OR focalin OR jornay OR adzenys OR methamphetamine OR desoxyn OR "methamphetamine s" OR methamphetamines OR dynavel OR evekeo OR mydayis OR qelbree OR viloxazin OR viloxazine OR psychotropic OR psychotropics OR psychostimulant OR psychostimulants OR psychopharmacological OR psychopharmacologicals OR medication OR medications OR pharmacological OR pharmacologics OR pharmacologically OR pharmacologic OR stimulant OR stimulants OR "non-stimulant" OR "non-stimulants") | 865,378 |
| 4 Anxiety | TS=("Anxiety Disorders" OR Anxiety OR anxieties OR "anxiety s" OR anxious OR anxiousness OR nervousness OR nervous OR worried OR worries OR worry OR worrying OR worrisome OR distress OR distressed OR distresses OR distressful OR distressing OR stress OR stressed OR stresses OR stressful OR stressfulness OR stressing OR uncertainty OR uncertainty OR insecure OR insecurely OR insecurities OR insecurity OR angst OR hypervigilance OR hypervigilant OR panic OR panic OR panics OR neurotic OR neurotically OR neurotics OR Neurosis OR Neuroses OR Psychoneuroses OR "Phobic Disorders" OR Phobia OR Phobic OR Agoraphobia OR "Panic Disorder") | 4,121,545 |
| 5 | #1 AND #2 AND #3 AND #4 | 3,115 |
| 6 study filter | TS=("case control study" OR “case control studies” OR "retrospective study" OR “retrospective studies” OR "controlled study" OR “control groups” OR "control group" OR "cohort analysis" OR "longitudinal study" OR "follow up" OR “follow-up” OR "prospective study" OR "retrospective study" OR "cross-sectional study" OR prevalence OR "prevalence study" OR lifespan OR (case AND control) OR (cases AND controlled) OR (cases AND comparison*) OR "control group" OR "control groups" OR cohort OR longitudinal OR prospective OR retrospective OR “cross-sectional” OR prevalence OR "transversal study" OR lifespan) | 5,849,442 |
| 7 | #5 AND #6 | 1,413 |
