# Supplementary material for: Does age of ADHD medication initiation predict long-term risk of anxiety? A scoping review
Source: PLOS Ment Health. 2025 Jan 10;2(1):e0000230. doi: 10.1371/journal.pmen.0000230 (PMC12798288; doi:10.1371/journal.pmen.0000230)
Supplement: S3 Table — (DOCX) [file pmen.0000230.s003.docx]

**S3 Table. Non-English Abstract Summaries**

| **Author (year)** | **Language and Title** | **Journal** | **Summary of methods and results** |
| --- | --- | --- | --- |
| Altomare et al. (2016) | *Italian* - Prescription monitoring methylphenidate and atomoxetine in the treatment of Attention Deficit Hyperactivity Disorder | Giornale Italiano di Farmacia Clinica | Secondary analysis of pharmaceutical data to examine appropriateness of ADHD prescriptions. Found that methylphenidate was more commonly prescribed, primarily in children ages 6-11, and ADHD prescriptions decreased during the summer months.  No mention of anxiety in the abstract. |
| Chambry et al. (2011) | *French* - Attention deficit disorder: multidisciplinary observational study over 1 year | L’Encéphale | 36 children with ADHD, with no prior treatment with methylphenidate, were evaluated at study enrollment and one year later. At follow-up, treated children demonstrated improved attention and executive function, but not improvement in anxiety. |
| GarcÍa GarcÍa et al. (2008) | *Spanish* - Attention deficit and hyperactivity disorder, a current problem | Anales de PediatrÍa | Aimed to describe clinical characteristics of children with ADHD at a single center. 83 children aged 3-8 were included. Methylphenidate was associated with positive outcomes.  No mention of anxiety in the abstract. |
| Garcia-Perez et al. (2005) | *Spanish* - The clinical semiology of attention deficit hyperactivity disorder according to age, and the effectiveness of treatments at different ages | Revista de Neurologia | Followed 152 children with suspected ADHD over 6 years, evaluating age at diagnosis and medication initiation. Older age at diagnosis and/or medication initiation was associated with improved outcomes. Outcomes included school performance and conduct disorders.  No mention of anxiety in the abstract. |
| Mardomingo Sanz et al. (2019) | *Spanish* - Assessment of comorbidity and social anxiety in adolescents with attention deficit hyperactivity disorder | Anales de PediatrÍa | Cross-sectional, observational study of adolescents aged 12-18 with ADHD. Adolescents with primarily inattentive presentation had higher levels of social anxiety.  Did not compare medicated vs unmedicated. |
| Montanes-Rada et al. (2009) | *French* - Drugs for attention deficit hyperactivity disorder | Revue Neurologique | Compared immediate- and extended-release methylphenidate and atomoxetine, describing the benefits and drawbacks of each.  No mention of anxiety in the abstract. |
| Salin-Pascual et al. (2018) | *Spanish* - Methylphenidate as an aid in patients with social anxiety receiving escitalopram | Revista Mexicana de Neurociencia | Experimental study comparing escitalopram alone vs escitalopram + methylphenidate in 15 patients with social anxiety. Apparent reduction in social anxiety was noted in participants receiving methylphenidate.  Age of participants is unclear based on the abstract. |
| Wiesegger et al. (2007) | *German* - Pharmacotherapy of attention-deficity/hyperactivity disorder (ADHD) and comorbid disorders | Neuropsychiatrie: Klinik, Diagnostik, Therapie und Rehabilitation: Organ der Gesellschaft Osterreichischer Nervenarzte und Psychiater | Presents algorithms which can serve as guidelines in selecting appropriate pharmacotherapy for patients with ADHD. Suggests atomoxetine can be used as first-line treatment in patients with comorbid anxiety disorders.  Type of article and age of participants are unclear based on the abstract. |
| Zavadenko & Suvorinova (2007) | *Russian* - Disorders comorbid to attention deficit hyperactivity syndrome in children | Zhurnal Nevrologii i Psikhiatrii Imeni S.S. Korsakova | Described the prevalence of comorbid disorders in 76 children aged 5-11 with ADHD. Cites evidence that atomoxetine is superior to placebo in treating children with ADHD and comorbid disorders.  Did not compare medicated vs unmedicated. |
| Zhang et al. (2006) | *Chinese* - Effect of electroencephalogram biofeedback on behavioral problems in children with attention deficit hyperactivity disorder | Chinese Journal of Clinical Rehabilitation | RCT comparing electroencephalogram (EEG) biofeedback and methylphenidate in 44 children with previously untreated ADHD. Participants were followed for 6 months. EEG biofeedback improved anxiety where methylphenidate did not. |
