# Supplementary material for: Does age of ADHD medication initiation predict long-term risk of anxiety? A scoping review
Source: PLOS Ment Health. 2025 Jan 10;2(1):e0000230. doi: 10.1371/journal.pmen.0000230 (PMC12798288; doi:10.1371/journal.pmen.0000230)
Supplement: Attachment — Submitted filename: Response to Reviewers.docx [file pmen.0000230.s004.docx]

November 22, 2024

Gellan Karamallah Ramadan Ahmed
Academic Editor
PLOS Mental Health

Dear Dr. Ahmed,

I would like to express my sincere gratitude to the reviewers for their time and their constructive feedback. We have taken this feedback into consideration and made changes, as described below.

We added the underlying data from Covidence, including screening decisions and data extraction, to the Duke Research Data Repository, which can be accessed at <https://doi.org/10.7924/r49p38q4j>. The data availability statement has been updated to reflect this change as well. An author summary has been included following the abstract, and further clarification has been added regarding the change in methodology, the rationale for database selection, and the exclusion of non-English articles during the full-text screening stage. An additional table has been added to the supplementary material describing the non-English articles that were excluded.

All typographical and grammatical errors were corrected, and the reference list was reviewed for completeness and correctness. Per submission guidelines, the PRISMA diagram was omitted from the text and included in the submission as a separate file.

Thank you again for your feedback and consideration.

Sincerely,

Margaret Fletcher, BSN, RN

PhD Student

Duke University School of Nursing

307 Trent Dr.

Durham, NC 27710

[margaret.fletcher@duke.edu](mailto:margaret.fletcher@duke.edu)
